# Combinatorial Herpes Simplex Vaccine Strategies: From Bedside to Bench and Back

**DOI:** 10.3389/fimmu.2022.849515

**Published:** 2022-04-25

**Authors:** Aziz A. Chentoufi, Nisha R. Dhanushkodi, Ruchi Srivastava, Swayam Prakash, Pierre-Gregoire A. Coulon, Latifa Zayou, Hawa Vahed, Hiba A. Chentoufi, Kathy K. Hormi-Carver, Lbachir BenMohamed

**Affiliations:** ^1^Laboratory of Cellular and Molecular Immunology, Gavin Herbert Eye Institute, School of Medicine, University of California Irvine, Irvine, CA, United States; ^2^Department of Vaccines and Immunotherapies, TechImmune, Limited Liability Company (LLC), University Lab Partners, Irvine, CA, United States; ^3^Biomedical Sciences, University of Ottawa, Ottawa, ON, Canada; ^4^Department of Molecular Biology & Biochemistry, Institute for Immunology, School of Medicine, University of California Irvine, Irvine, CA, United States

**Keywords:** herpes simplex virus, clinical trials, vaccines, asymptomatic, immune checkpoint blockade

## Abstract

The development of vaccines against herpes simplex virus type 1 and type 2 (HSV1 and HSV-2) is an important goal for global health. In this review we reexamined (*i*) the status of ocular herpes vaccines in clinical trials; and (*ii*) discusses the recent scientific advances in the understanding of differential immune response between HSV infected asymptomatic and symptomatic individuals that form the basis for the new combinatorial vaccine strategies targeting HSV; and (*iii*) shed light on our novel “asymptomatic” herpes approach based on protective immune mechanisms in seropositive asymptomatic individuals who are “naturally” protected from recurrent herpetic diseases. We previously reported that phenotypically and functionally distinct HSV-specific memory CD8^+^ T cell subsets in asymptomatic and symptomatic HSV-infected individuals. Moreover, a better protection induced following a prime/pull vaccine approach that consists of first priming anti-viral effector memory T cells systemically and then pulling them to the sites of virus reactivation (e.g., sensory ganglia) and replication (e.g., eyes and vaginal mucosa), following mucosal administration of vectors expressing T cell-attracting chemokines. In addition, we reported that a combination of prime/pull vaccine approach with approaches to reverse T cell exhaustion led to even better protection against herpes infection and disease. Blocking PD-1, LAG-3, TIGIT and/or TIM-3 immune checkpoint pathways helped in restoring the function of antiviral HSV-specific CD8^+^ T cells in latently infected ganglia and increased efficacy and longevity of the prime/pull herpes vaccine. We discussed that a prime/pull vaccine strategy that use of asymptomatic epitopes, combined with immune checkpoint blockade would prove to be a successful herpes vaccine approach.

## Introduction

According to the World Health Organization (WHO), over two-thirds of the worldwide population in infected with HSV-1 (commonly known to cause oral herpes or cold sores) and HSV-2 (commonly known to cause genital herpes) (1, 2). The prevalence of HSV-1 and HSV-2 is 47.8% and 11.9%, respectively, for individuals aged 14 to 49 years according to a 2018 February data brief published by the US Centers for Disease Control and Prevention’s National Center for Health Statistics (1, 2). In the United States alone, every year, there are 500,000 HSV-1 oral herpes cases; 300,000 HSV-1 and HSV-2 genital herpes cases; 20,000 HSV-1 ocular herpes cases and 1,500 cases of herpes encephalitis (3, 4). Apart from being the most prevalent sexually transmitted disease, HSV-1 is the leading cause of infectious blindness in Western countries (5). HSV-1 and HSV-2 are neurotropic viruses that infect the anogenital, oral mucosal lining and the skin and the eyes (6) The immune response to HSV typically controls the acute mucosal infection; however, the virus remains latent in the ganglia, and there is a life-long sporadic low-grade shedding of virus from sensory neurons into the mucosa (6). Thus, while HSV hides for a lifetime in the trigeminal, autonomic, or dorsal root ganglia, it reactivates and sheds asymptomatically making the transmission high. In addition to causing painful blisters, HSV-2 can cause encephalitis and death in newborns from vertical transmission and increases the risk for HIV infection two-three-fold times (7). Antiviral drugs are the only current treatment approved by the Food and Drug Administration (FDA) for treatment of herpetic diseases. Due to the cost, virus resistances and limited effectiveness of antiviral drugs, preventive or therapeutic vaccines are highly desirable to control herpes infection and/or diseases (8). The development of a vaccine that proves effective against one type of the HSV would be helpful for the other type due to the genetic similarity between HSV-1 and HSV-2. However, due to virus latency and HSV immune evasion, immunotherapy and vaccine development against the virus have become a real challenge. As of 2018, a number of different HSV vaccine candidates were at different stages of clinical trials (9, 10) (**Table 1**).

**Table 1:** Herpes Vaccine Strategies.

Type of Vaccine	Vaccine Construct	Administration Route	Phase ofTrial	Virus Subtype	Results	Limitations	Ref.
**Inactivated vaccine**	**HSV-1 gH deletion (SC16∆gH)**	Subcutaneous in human	Clinical trial	HSV-2	Unable to show protection against acute or recurrent genital herpes infection Does not show improvement in recurrences and disease severity. Does not affect on viral shedding	Vaccine did not achieve clinical usefulness Alternative approaches could be proposed	(11) Akhrameyeva NV, Zhang P, Sugiyama N, Behar SM, Yao F. Development of a glycoprotein D- expressing dominant- negative and replication- defective herpes simplex virus 2 (HSV-2) recombinant viral vaccine against HSV-2 infection in mice. *J Virol*, 85(10), 5036-5047 (2011).
		Subcutaneous and intravaginal in guinea pig	Preclinical trial	HSV-2	Provides complete protection against primary and recurrent HSV infection Induces high neutralizing antibody titers Induces long- lasting immune responses i.e., over 6 months Develops high potency for complete HSV protection	Missing reproducibility on correlation between antibody titers and recurrent infection pattern The immune mechanisms involved in the control of recurrent infection need to be elucidated	(12) Reszka NJ, Dudek T, Knipe DM. Construction, and properties of a herpes simplex virus 2 dl5-29 vaccine candidate strain encoding an HSV-1 virion host shutoff protein. *Vaccine*, 28(15), 2754-2762 (2010)
		Intraepithelial and intravaginal in guinea pig	Preclinical trial	HSV-2	Reduces HSV symptoms Gives quicker symptomatic episodes Prevents local HSV-2 replication offers Improved protection against HSV severity via Intravaginal route	High risk of genetic recombination Unable to block the virus reactivation to prevent disease recurrences This study needs more animal experiment for statistical significance	(13) Belshe PB, Leone PA, Bernstein DI *et al.* Efficacy Results of a Trial of a Herpes Simplex Vaccine. *The New England journal of medicine*, 366, 34-43 (2012).
		Scarification via ear pinna route in mice	Preclinical trial	HSV-1	Establishes self-limiting HSV infection Induces DTH response Provides protection against acute HSV infection	May reactivate latent HSV Viral latency and reactivation should be studied in more suitable animal model	(14) Bernard MC, Barban V, Pradezynski F *et al.* Immunogenicity, protective efficacy, and non-replicative status of the HSV-2 vaccine candidate HSV529 in mice and guinea pigs. *PLoS One*, 10(4), e0121518 (2015).
	**HSV-2 ICP8 replication defective + B7 co- stimulation**	Subcutaneous in mice	Preclinical trial	HSV-2	Increases IFN-g-producing T- cells Decreases HSV replication in genital mucosa Lowers HSV related genital and neurological disease Reduces mortality	The protective immunity mediated by antibody and T- cells	(15, 16) Ohashi M, Bertke AS, Patel A, Krause PR. Spread of herpes simplex virus to the spinal cord is independent of spread to dorsal root ganglia. *J Virol*, 85(6), 3030-3032 (2011). Dasgupta G, Chentoufi AA, Kalantari M *et al.* Immunodominant "asymptomatic" herpes simplex virus 1 and 2 protein antigens identified by probing whole-ORFome microarrays with serum antibodies from seropositive asymptomatic versus symptomatic individuals. *J Virol*, 86(8), 4358-4369 (2012).
	**Multiple genes Deletion of HSV-2**	Subcutaneous in mice	Preclinical trial	HSV-2	Reduces viral titer and viral shedding Suppreses viral replication and latency Theorotically provides protection against double- mutant virus even in immunocompro mised individuals	The genetic basis underlying the latency defect should be elucidated	(17) Dasgupta G, Nesburn AB, Wechsler SL, BenMohamed L. Developing an asymptomatic mucosal herpes vaccine: the present and the future. *Future Microbiol*, 5(1), 1-4 (2010).
	**HSV-2 ICP10∆PK deletion**	Subcutaneous in mice	Preclinical trial	HSV-2	Induces memory T-cells and establish strong T-helper type 1 (Th1) immune response Increases IL-12 secretion by DCs	Does not readily begin latency Must show the frequency and duration of memory T-cells Assess the ability to activate p38MAPK in T- cells	(18) Chentoufi AA, BenMohamed L. Future viral vectors for the delivery of asymptomatic herpes epitope-based immunotherapeutic vaccines. *Future virology*, 5(5), 525-528 (2010).
	**HSV-2 UL5 & UL29 genes deletion**	Intramuscular in humans	Clinical trial	Multiple mutated HSV-1 and HSV-2 combina tions	Safe and well tolerated Produces neutralizing antibody along with CD4+ and CD8+ T-cell responses in HSV seronegative individuals Produces only CD4+ T-cell responses in HSV seropositive individuals	More reactions than placebo on the injection site Should modify vaccine by increasing the expression of certain viral proteins Should inhibits the expression of viral immune evasion genes, or adding an adjuvant	(19) Schiffer JT, Abu-Raddad L, Mark KE *et al.* Mucosal host immune response predicts the severity and duration of herpes simplex virus-2 genital tract shedding episodes. *Proc Natl Acad Sci U. S. A.*, 107(44), 18973-18978 (2010).
		Subcutaneous, and intramuscular in mice	Preclinical trial	HSV-2	Decreases genital infection and viral shedding Produces strong immune response Gives protection against many HSV-2 viral strains Shows better protection via intramuscular route	Should study the role and type of DC involved in priming immunity against the intramuscular vaccine	(20) Chentoufi AA, Binder NR, Berka N *et al.* Asymptomatic human CD4+ cytotoxic T-cell epitopes identified from herpes simplex virus glycoprotein B. *J Virol*, 82(23), 11792-11802 (2008).
	**HSV-2 gD (∆gD-2) deletion**	Intramuscular in mice	Preclinical trial	HSV-2 and superin- fection (HSV-1^+^)	Induces IgG2 response Fully protects HSV-2 spreading to the sacral ganglia and mortality Shows almost no signs of disease	voir in the Should use guinea pigs as an animal model to study recurrent diseases Should incorporate murine superinfection model in preclinical evaluation of HSV- vaccine candidates	(21) Dervillez X, Qureshi H, Chentoufi AA *et al.* “Asymptomatic” HLA- A*02:01-Restricted Epitopes from Herpes Simplex Virus Glycoprotein B Preferentially Recall Polyfunctional CD8+ T Cells from Seropositive Asymptomatic Individuals and Protect HLA Transgenic Mice Against Ocular Herpes. *J Immunol*, (2013).
**Live attenuated vaccine**	**R7017 Deletion of HSV-1 thymidine kinase**	Intracerebral in mice, vaginal, intradermal, and intramuscular in guinea pigs and scarification of cornea in rabbits	Preclinical trial	HSV-1 and HSV-2	Protects against severe HSV infections HSV lesions are localized, superficial and heals more rapidly	It establishes low frequency of latent infections in all hosts (R7020) It also establishes latent infection in rabbits (R7017)	(22) Dervillez X, Gottimukkala C, Kabbara KW *et al.* Future of an "Asymptomatic" T-cell Epitope-Based Therapeutic Herpes Simplex Vaccine. *Future virology*, 7(4), 371-378 (2012).
	**RAV9395 (Deletion of HSV-2 γ134.5 gene, UL55 and UL56 ORF)**	Intramuscular	Preclinical trial	HSV-2	Decreases lesion development and HSV infection severity Decreases frequency of HSV reactivation from explanted DRG	N/A	(23) Pope C, Kim SK, Marzo A *et al.* Organ- specific regulation of the CD8 T cell response to Listeria monocytogenes infection. *Journal of immunology*, 166(5), 3402-3409 (2001).
	**VC2 (mutations in gK and UL20)**	Intramuscular	Preclinical trial	HSV-1 and HSV-2	Fully protects against lethal intravaginal HSV challenge Presents cross-protective humoral and cellular immunity Absence of viral DNA in ganglionic tissues	N/A	(24) Gebhardt T, Whitney PG, Zaid A *et al.* Different patterns of peripheral migration by memory CD4+ and CD8+ T cells. *Nature*, 477(7363), 216-219 (2011).
		Intramuscular	Preclinical trial	HSV-2	Decreases acute viral replication in vagina, amount of virus in neural tissue, subsequent recurrent disease, and viral shedding Delivers protection after 6 months	Applying the criteria used for human trials	(25) Nelson MH, Bird MD, Chu CF *et al.* Rapid clearance of herpes simplex virus type 2 by CD8+ T cells requires high level expression of effector T cell functions. *J Reprod Immunol*, 89(1), 10-17 (2011).
	**HSV-2 ICP0-∆NLS)**	Footpad injection	Preclinical trial	HSV-2	Significantly reduces viral shedding in vagina No detectable infection	N/A	(26) Bertke AS, Patel A, Imai Y, Apakupakul K, Margolis TP, Krause PR. Latency-associated transcript (LAT) exon 1 controls herpes simplex virus species-specific phenotypes: reactivation in the guinea pig genital model and neuron subtype-specific latent expression of LAT. *J Virol*, 83(19), 10007-10015 (2009).
	**HSV-2 gE deletion**	Intramuscular, intravaginal, and intravenous	Preclinical trial	HSV-2	No disease mortality Absence of infectious virus in DRG and recurrent HSV shedding in vagina Decreases recurrent genital HSV lesions Gives better efficacy through intramuscular route than subcutaneous route	Provides incomplete protection	(27) Schiffer JT, Corey L. Rapid host immune response and viral dynamics in herpes simplex virus-2 infection. *Nat Med*, 19(3), 280-290 (2013).
	**VC2 (gKD31-68 deletion of HSV-1)**	Intramuscular	Preclinical trial	HSV-2	Shows poor HSV replication at the immunization site Rarely infects neural tissue Lack of any genital disease Reduces severity of acute and recurrent HSV-2 shedding in vagina and quantity of virus in DRG Better selection as a prophylactic vaccine	Not effective as a therapeutic vaccine	(28) Tang VA, Rosenthal KL. Intravaginal infection with herpes simplex virus type-2 (HSV-2) generates a functional effector memory T cell population that persists in the murine genital tract. *J Reprod Immunol*, 87(1-2), 39-44 (2010).
		Intramuscular	Preclinical trial	HSV-1	Gives protection against HSV-1- induced ocular pathogenesis Provides complete recovery from initial conjunctivitis Increases neutralizing antibody titers along with CD3+, CD4+ and CD8+ T-cells Decreases infiltration of Iba1+ macrophages	N/A	(29) van Lint A, Ayers M, Brooks AG, Coles RM, Heath WR, Carbone FR. Herpes simplex virus specific CD8+ T cells can clear established lytic infections from skin and nerves and can partially limit the early spread of virus after cutaneous inoculation. *J Immunol*, 172(1), 392-397 (2004).
	**R2 (HSV-1 mutation in region 2 of pUL37**)	Intramuscular, intradermal, and intravaginal	Preclinical trial	HSV-2	Increases neutralizing antibodies Decreases acute and recurrent HSV latent virus detection in DRG and recurrent shedding Rarely infects neural tissue Shows more effectivity via intradermal route	N/A	(30) Rott LS, Briskin MJ, Andrew DP, Berg EL, Butcher EC. A fundamental subdivision of circulating lymphocytes defined by adhesion to mucosal addressin cell adhesion molecule 1. Comparison with vascular cell adhesion molecule-1 and correlation with beta 7 integrins and memory differentiation. *J Immunol*, 156(10), 3727-3736 (1996).
	**HSV-1 ICP0∆NLS**	Subcutaneous and intramuscular	Preclinical trial	HSV-1	Shows less infectious virus during acute infection in TG and brainstem Stimulates an immune response by increasing the gB-elicited interferon (IFN)- γ, granzyme B and CD107a; and decreasing LAG-3, PD-1, and TIM-3 Gives protection against ocular HSV-1 challenge by reducing ocular neovascularization and suppressing peripheral nerve virus replication	T-cell response is only observed at a single time point	(31) Mebius RE, Streeter PR, Michie S, Butcher EC, Weissman IL. A developmental switch in lymphocyte homing receptor and endothelial vascular addressin expression regulates lymphocyte homing and permits CD4+ CD3- cells to colonize lymph nodes. *Proc Natl Acad Sci U S A*, 93(20), 11019-11024 (1996).
**Naked DNA vaccine**	**pSVL- HSV-1 gD, pRc/CMV- HSV-1 gD**	Intramuscular	Preclinical trial	HSV-1	Reduces serum anti-gD antibody, anti-HSV1 neutralizing antibody and anti-gD ELISA responses Gives non- specific changes in ELISA and neutralization antibody titers	Provides low protection against HSV-1 Not a useful alternative of a gD subunit vaccine	(32) Mackay CR, Andrew DP, Briskin M, Ringler DJ, Butcher EC. Phenotype, and migration properties of three major subsets of tissue homing T cells in sheep. *Eur J Immunol*, 26(10), 2433-2439 (1996).
	**pDNA encoding HSV-2 gD2**	Intramuscular	Clinicaltrial	HSV-1-/HSV-2-, HSV-1+/HSV-2-	Provides safe and well tolerated with no dose-limiting toxicities Increases D2-specific cytotoxic T- cell and lymphoproliferati on immune responses	Produces adverse events that are mostly local site reactions	(33) Abitorabi MA, Mackay CR, Jerome EH, Osorio O, Butcher EC, Erle DJ. Differential expression of homing molecules on recirculating lymphocytes from sheep gut, peripheral, and lung lymph. *J Immunol*, 156(9), 3111-3117 (1996).
	**pDNAs encoding HSV-2 gD2**	Subcutaneous	Preclinical trial	HSV-2	Provides fully protection against lethal intravaginal HSV-2 infection Produces strong HSV-2 virion- specific IgG and neutralizing antibody responses Reduces all levels of recurrent HSV-2 significantly Reduces acute and recurrent disease, recurrent lesion days and latent HSV-2 load	Should be studied in a greater number of guinea pigs	(34) von Andrian UH, Mackay CR. T-cell function and migration. Two sides of the same coin. *N Engl J Med*, 343(14), 1020-1034 (2000).
	**pDNA encoding HSV-2 gD2 coupled with Vaxfectin ®**	Intramuscular	Preclinical trial	HSV-2	Increases IgG antibody titers Provides protection against lethal HSV-2 challenge Reduces vaginal HSV load and viral latency in DRG	Limited sensitivity for IgG assay	(35) Mackay LK, Wakim L, van Vliet CJ *et al.* Maintenance of T cell function in the face of chronic antigen stimulation and repeated reactivation for a latent virus infection. *J Immunol*, 188(5), 2173-2178 (2012).
	**pDNA encoding HSV-2 gD2 and UL46 and UL47 genes coupled with Vaxfectin ®**	Intramuscular	Preclinical trial	HSV-2	Reduces viral replication and shedding in genital tract, latent HSV-2 DNA in DRG, and frequency of recurrent disease Completely protects from both primary and recurrent genital disease	Includes additional controls including irrelevant plasmids coupled with Vaxfectin®	(35) Mackay LK, Wakim L, van Vliet CJ *et al.* Maintenance of T Cell Function in the Face of Chronic Antigen Stimulation and Repeated Reactivation for a Latent Virus Infection. *J Immunol*, (2012).
	**Codon-modified polynucleo-tide vaccine**	Intradermal in forearm	Clinical trial	HSV-2	Provides safe and well tolerated protection with no moderate or serious adverse effects Increases immune cellular activity	Minimal antibodies increase with overall no statistical significance Insufficient number of subjects to determine a significant placebo effect	(36) Mackay LK, Stock AT, Ma JZ *et al.* Long-lived epithelial immunity by tissue-resident memory T (TRM) cells in the absence of persisting local antigen presentation. *Proc Natl Acad Sci U S A*, 109(18), 7037-7042 (2012)
	**COR-1: (1) Full-length HSV-2 envelope gD2 and (2) truncated version of gD2 fused to a ubiquitin sequence**				Presence of CD45^+^, CD4^+^, CD68^+^ macrophages and polymorphonucle ar neutrophils at site of immunization Decreases mean number of outbreaks and viral shedding		
	**SLV-20: (1) pGX27 with tissue plasmino- gen activator (tpa), Flt3L and HSV-2 gB and UL39, (2) pGX27 with gD2, ICP0 and ICP4 and (3) pGX27 with IL-12- IL-21 and MIP-1α**	Intramuscular	Preclinical trial	HSV-2	Inhibits pathological progression after viral infection Increases survival rate Reduces virus titer and viral shedding Increases IFN- γ, CD4+, CD8+ and CD44hiCD62Lhi central memory T-cells expression	Does not show any significant differences in immunoglobulin IgA, IgM, IgG1 and IgG3 levels	(37) Masopust D, Picker LJ. Hidden memories: frontline memory T cells and early pathogen interception. *J Immunol*, 188(12), 5811-5817 (2012).
**Protein- based subunit vaccine**	**HSV-2 gD2t with 3-O-deacylated mono- phosphoryl**	Intramuscular	Preclinical trial	HSV-1	Reduces latent viral load significantly Provides protection against acute and recurrent HSV-2 infection	Not as effective as replication- defective *dl5-29*	(38) Suni MA, Ghanekar SA, Houck DW *et al.* CD4(+) CD8(dim) T lymphocytes exhibit enhanced cytokine expression, proliferation, and cytotoxic activity in response to HCMV and HIV-1 antigens. *Eur J Immunol*, 31(8), 2512-2520 (2001).
	**lipid A (MPL)- aluminum hydroxide (alum)**	Subcutaneous	Preclinical trial	HSV-2	Provides protection against acute and recurrent HSV infection and acute viral shedding Reduces recurrent lesion days; sufficient to prevent most recurrent lesion episodes significantly	Does not show significant reduction in the mean number of days with recurrent diseases Not sufficient to suppress early stages of viral reactivation Produces low levels of HSV-2 virion-specific antibodies	(34) von Andrian UH, Mackay CR. T-cell function and migration. Two sides of the same coin. *N Engl J Med*, 343(14), 1020-1034 (2000).
	**HSV-2 gD with MPL- alum**	Intramuscular	Clinical trial	HSV-1-/HSV-2-, HSV-1^±^/HSV-2^±^	Presents a protective effect in those women who were HSV-1 and HSV-2 seronegative	Ineffective in women who are seropositive for HSV-1 but seronegative for HSV-2 Ineffective in men regardless of serologic status	(39) Jiang X, Chentoufi AA, Hsiang C *et al.* The herpes simplex virus type 1 latency associated transcript (LAT) can protect neuronal derived C1300 and Neuro2A cells from Granzyme B induced apoptosis and CD8 T-cell killing. *J Virol*, (2010).
		Subcutaneous	Preclinical trial	HSV- 1 and HSV- 2	Gives almost complete protection against primary infection Presents better protection against latent infection	Does not prevent mucosal infection	(40)
	**HSV-2 gD and gB adjuvanted with a novel T- cell antigen and tegument protein UL40**	Intramuscular	Preclinical trial	HSV-2	Increases HSV-2 antigen-specific CD8+ T- cell responses Stimulates high titers of neutralizing antibodies Reduces HSV shedding in vagina, lesion scores and latent infection	N/A	(41) Jameson SC, Masopust D. Diversity in T cell memory: an embarrassment of riches. *Immunity*, 31(6), 859-871 (2009).
	**HSV-2 gD2 and gB2 formulated in a nano- emulsion adjuvant (NE01- gD2/gB2)**	Intranasal and intramuscular	Preclinical trial	HSV-2	Increases neutralizing antibodies levels Reduces acute and recurrent disease scores and shedding of virus Reduces detection of latent virus in DRG	Less efficiently induces neutralizing antibodies than intramuscular IgD2 with MPL- alum vaccine	(42) Khan AA, Srivastava R, Spencer D *et al.* Phenotypic and Functional Characterization of Herpes Simplex Virus Glycoprotein B Epitope-specific Effector and Memory CD8+ T Cells from Ocular Herpes Symptomatic and Asymptomatic Individuals. *Journal of virology*, (2015).
	**Trivalent (gC2, gD2, gE2) subunit vaccine mixed with CpG and alum**	Intramuscular	Preclinical trial	HSV-2	Produces antibodies that binds to gC2 and blocks its ability to bind C3b for immune evasion	gC2 are not immunogenic Without adjuvant during natural HSV-2 infection in humans or HSV-2 infected guinea pigs	(43) Shin H, Iwasaki A. A vaccine strategy that protects against genital herpes by establishing local memory T cells. *Nature*, 491(7424), 463-467 (2012).
		Intramuscular	Preclinical trial	HSV-1 and HSV-2	Increases HSV glycoprotein- specific antibodies which neutralizes HSV-1 and HSV-2 Provides remarkable durability of vaccine response (continues up to 21 months post- immunization) Exhibits little to no viral replication Absence of viral DNA in brains or trigeminal ganglia Provides protection against nHSV (maternal immunization promotes transfer of neutralizing antibodies and protects offspring from disseminated disease, weight loss, anxiety-like behaviour, and mortality)	N/A	(44) Khan AA, Srivastava R, Chentoufi AA *et al.* Bolstering the Number and Function of HSV-1-Specific CD8(+) Effector Memory T Cells and Tissue-Resident Memory T Cells in Latently Infected Trigeminal Ganglia Reduces Recurrent Ocular Herpes Infection and Disease. *J Immunol*, 199(1), 186-203 (2017).

One common denominator in these vaccines is the use of the whole virus or whole virus proteins, which contain both protective “asymptomatic” epitopes and pathogenic “symptomatic” epitopes. Our developed “asymptomatic” herpes vaccine approach which is based on understanding the immune mechanisms by which seropositive asymptomatic individuals are “naturally” protected from recurrent herpes disease throughout their lives. Clinical and pre-clinical studies have proved that the T cell-based immune system in the mucosa lining of the genital tract plays a crucial role in the prevention of HSV acquisition. A better mucosal vaccine approach to boost effector memory T cell responses will serve instrumental in developing an effective HSV vaccine (45). Our latest approach of using adenoviral vectors delivering chemokines and asymptomatic dominant epitopes to induce and pull antiviral CD4^+^ and CD8^+^ T cells to the site of reactivation (i.e., ganglia) and replication (i.e., epithelia) would be an effective combinatorial herpes simplex vaccine strategy. Moreover, another combinatorial herpes simplex vaccine strategy that consists of reversing T cell exhaustion by immune checkpoint blockade would be a successful strategy to clear herpes infection (46). In this review, we highlight the current clinical trials in herpes vaccine development and emphasize the significance of using the asymptomatic epitope approach in a combinatorial vaccine strategy.

## HSV Vaccines: From Past to Present

The success of vaccines against other alpha herpes, like the chicken-pox and shingles vaccine, has given hope for the development of a vaccine against HSV (47) (**Table 1**). Four main vaccine approaches have been designed and tested in the past four decades to fight off herpes simplex virus type 1 (HSV-1) and type 2 (HSV-2) infections and diseases (48): (1) Inactivated “killed” HSV vaccines; (2) Live-attenuated HSV vaccines; (3) Replication-defective HSV vaccines; and (4) Subunit HSV vaccines (9, 49–54). Each of these types of vaccine approaches has its pros and cons when it comes to safety, immunogenicity, and protective efficacy.

### Inactivated “Killed” HSV Vaccines

HSV is a highly successful neurotropic virus that resides in the nervous system and therefore presents the risk of developing neuro-pathogenesis and life-threatening Herpes Simplex Encephalitis (HSE). Thus, back in the 70s and 80s, the first whole inactivated HSV vaccine approach used “kill” the whole virus after exposure to heat, UV-light (55) or chemicals (56, 57). These whole inactivated HSV vaccines induced antibodies, but not T cells, and as such have not been successful in the protection against recurrent HSV-1 or HSV-2 infections and diseases (58–60). Therefore, the live-attenuated HSV vaccines (61–66) and replication-defective HSV vaccines were introduced (51, 58–60, 67–71).

### Live-Attenuated HSV Vaccines

Live-attenuated HSV vaccines contrast inactivated HSV vaccines produced by “killing” the virus and reducing the neurovirulence of HSV-1 or HSV-2, while keeping them viable. In the past 24 years, many live-attenuated HSV vaccines have been introduced and tested in both the mouse and guinea pig models mainly in a prophylactic setting (instead of a therapeutic setting). However, due mostly to safety concerns, only a few of these live vaccines have progressed into clinical trials (63). Live-attenuated HSV vaccines include: (**1**) The HSV-2 TK^(-)^ mutant reported back in 1995 by Milligan and Bernstein and then by Kiyono in 2014 (72); (**2**) the RAV 9395 live attenuated recombinant virus; evaluated in guinea pigs and reported by Spaete back in 1998 (70); (**3**) AD472, a live attenuated recombinant HSV-2 vaccine evaluated in guinea pigs was reported back in 2005 (51); (**4**) The most studied HSV-1 and HSV-2 ICP0 ^(-)^ live-attenuated mutant vaccines, lacking the nuclear localization signal (NLS) on the *ICP0* gene (0DeltaNLS), developed in 2010 by Halford and tested in mice and guinea pigs (69, 73–76); (**5**) The HSV2-gD27 mutant vaccine reported by Cohen in 2012 (77); (**6**) The HSV-2 gE2-del mutant vaccine reported by Friedman in 2012 (78); (**7**) The HSV-2 UL24 mutant tested in mice and guinea pigs reported by Visalli in 2014 (67); and (**8**) The HSV-1 VC2 mutant reported by Kousoulas in 2014 (79).

### Replication-Defective HSV Vaccines

Replication-defective virus vaccines, also called DISC (Disabled Infectious Single Cycle) virus vaccines, are defective for one or more genes that are essential for viral genome replication or synthesis and assembly of viral particles. In normal cells, they express viral gene products but do not replicate to form progeny virions. Replication-defective HSV vaccines can stimulate immune responses but produce no progeny viral particles. However, because they do not replicate and spread in the host, replication-defective virus vaccines may be less immunogenic, specifically less T cell stimulators because they have a relatively limited capacity to solicit professional antigen presenting cells (i.e., B, macrophage, and dendritic cells), a prerequisite for the induction of CD4^+^ and CD8^+^ T cell responses.

The replication-defective HSV vaccines developed during the last 24 years include: (**1**) DISC HSV-1 vaccine tested in guinea pigs by McLean, back in 1996 (80); (**2**) This was followed by another DISC HSV-2 vaccines which consisted of gH-deleted HSV-2 mutant tested in guinea pigs for recurrent genital herpes and reported by McLean in 1997 (81); (**3**) The HSV-2 mutant engineered by Dr. Knipe back in 1997, by replacing the *ICP8* gene of HSV-2 strain 186 with an *ICP8-lacZ* fusion gene from the HSV-1 HD-2 mutant strain. The resulting HSV-2 5BlacZ mutant was later tested in guinea pigs by the same group as reported in 2001 (61, 62), (**4**) The most studied replication-defective virus HSV-2 dl5-29 vaccine, was developed by Knipe in 2008 and tested in mice and guinea pigs by Cohen in 2010 (12, 59, 63, 82) and by Londono-Hayes in 2015 (14) and shown to be have a protective effect. Eventually, this vaccine progressed to human trials only to show unsuccessful results in a Phase 1 clinical trial conducted recently by Sanofi Pasteur; (**5**) The HSV-2 ACAM529 mutant tested in a mouse model of genital herpes challenge and reported by Knipe and others in 2010 and 2012 (12, 83, 84); (**6**) The HSV-1 Δ gK mutant tested in mouse model of herpes challenge and reported in 2013 by Kousoulas (85); (**7**) The HSV-1 CJ2-gD2 vaccine, a glycoprotein D-expressing replication-defective and dominant-negative HSV-1 recombinant viral vaccine, tested in mice guinea pigs and reported in 2011 (11) and 2014 by Yao (86); (**8**) The latest replication defective HSV vaccine is the HSV-2 ΔgD (gD1^-/+^) reported in 2015 by Herold and Jacobs group as being protective in a mouse model of genital herpes challenge (87). The efficacy of the HSV-2 ΔgD vaccine in prophylactic and therapeutic settings has yet to be evaluated in the guinea pig model of primary and recurrent genital herpes. Compared to clinical trials using adjuvanted subunit vaccines (e.g., the adjuvanted gD/gB vaccine trials), many live attenuated/replication defective vaccines-based Phase 1 trial trials, were either terminated or did not progress to Phase II, because of: (*i*) A lack of immunogenicity; and/or (*ii*) Concerns related to safety of using a live virus as vaccine, as detailed above.

### Subunit HSV Vaccines

A variety of subunit HSV vaccine approaches have been developed including proteins, DNA and peptide epitope-based vaccines (88, 89). Traditional protein-based vaccines are safe compared to live-attenuated and replication-defective HSV vaccines. Recombinant soluble HSV-2 glycoprotein D (gD) has been the most promising subunit vaccine that went into extensive clinical evaluation. Over the past 25 years, there has been one Phase II therapeutic genital herpes vaccine and three Phase III clinical trials of prophylactic subunit vaccines, all using the HSV-2 gD (or mixed with gB in one trial) (90–95). Back in 1994, the first therapeutic vaccine trial delivered the gD with aluminum salt (i.e. Alum) adjuvant in 98 symptomatic genital herpes patients who reported 4 to 14 recurrences per year (96). Unfortunately, this vaccine reduced the frequency of recurrences by only 24% despite that the vaccine boosted neutralizing antibodies to HSV-2 four-fold over baseline levels (96). These disappointing results from the first therapeutic gD/Alum vaccine trial suggested that for therapeutic protection; a vaccine must: (**1**) Induce CD4^+^ and CD8^+^ T cell responses, in addition to neutralizing antibodies, (**2**) Incorporate HSV-2 antigens other than gD; and (**3**) Must test different adjuvants, other than Alum. Three years later in 1997, the Chiron vaccine trial used a combination of gD and gB delivered together with the MF59 Novartis’ adjuvant, an oil-in-water emulsion of squalene oil, using the same target population of genital herpes patients as in the 1994 trial. This gB/gD/MF59 vaccine did not elicit T cell responses, produced high levels of neutralizing antibody to HSV-2, yet had only a 9% efficacy (94). This trial suggested that: (**1**) besides neutralizing antibodies, a protective vaccine must induce antiviral CD4^+^ and CD8^+^ T cell responses; (**2**) a therapeutic vaccine must incorporate HSV-2 antigens other than gB and gD; and (**3**) must test different adjuvants, other than Alum and MF59. Later, two GlaxoSmithKline (GSK) vaccine trials (one reported in 2004 and the other in 2012), used the gD protein delivered together with a different adjuvant, the 3-*0*-deacylated monophosphoryl lipid A (MPL), a TLR4 agonist (93) together with Alum (gD/MPL/Alum vaccine). The first trial enrolled discordant couples, who have regular partners with genital herpes, while the second trial enrolled HSV seronegative women who have multiple and random partners (93). The first trial, reported in 2004, showed a 74% efficacy against genital herpes disease caused by HSV-2 (93). Unfortunately, later, results using the same gD/MPL/Alum vaccine reported in 2012, showed only 58% efficacy against genital HSV-2 disease (13). The apparent contradictions in efficacy against genital HSV-2 disease, of the two GSK trials that used the same gD/MPL/Alum vaccine, is puzzling. The difference in efficacy in the two clinical trials attributed to different populations enrolled in each trial (i.e. discordant couples vs. random seropositive women with multiple partners) (13). In the first clinical trial, the distinguishing feature of discordant couples was that they were a highly selected group in which the uninfected partner is potentially repeatedly exposed to HSV by the infected partner. This likely increased risk of infection and disease, hence lowering the threshold of seeing a significant effect of the therapeutic vaccine. In other words, the attack rates of HSV-2 genital disease were high among discordant couples making easy to see a significant reduction following therapeutic vaccination. In contrast, the second clinical trial that enrolled random seropositive women, with multiple lifetime sexual partners, in which the attack rate and the risk of infection and disease was much lower and hence likely raised the threshold of seeing a significant effect of the therapeutic vaccine. Regardless of the targeted population, the first GSK vaccine trial that produced 74% protective efficacy also stimulated both T cells and neutralizing antibodies (13). In 2016-2018, a Genocea vaccine trial (designated as Gen-003) used a combination of ICP4 and gD2 truncated proteins with a novel adjuvant, named Matrix M-2 (MM-2) (89). Matrix M is a saponin-based adjuvant that has a balanced B and T cell immuno-stimulatory profile. This trial reported a significant reduction of recurrent herpes lesions and genital viral shedding (90–92). This protection appeared to correlate with blood-derived antiviral CD4^+^ and CD8^+^ T cell responses (90–92). Due to ethical and practical limitations, none of the vaccine clinical trials have investigated the local tissue resident CD4^+^ and CD8^+^ T cells in dorsal root ganglia (DRG) and vaginal mucosal tissues.

## Modified RNA (mRNA) Vaccine Platforms Against HSV-1 and HSV-2

RNA vaccines, during the current pandemic, have emerged as a versatile approach against emerging viral infections to overcome the challenges confronted with the conventional vaccine strategies ^1–7^. mRNA is the carrier of the genetic information necessary for the endogenous proteins synthesis, it does not integrate into the genome and safely metabolized and eliminated by the cells ^8–10^. RNA-based vaccines have been shown safe in animal models and in human clinical trials and trigger a strong innate immune response. Many strategies have been used to increase the delivery and immunogenicity of mRNA while diminishing innate immune sensing^11^. Free and protamine-complexed mRNA were among the first approaches to provide robust antigen expression and immune-stimulation ^12–14^. This vaccine set-up showed the ability to induce strong immunity and protective efficacy against lethal influenza or rabies viral infections in many animal models ^4,15^. The first ever prophylactic mRNA-based vaccine (CV7201) in healthy human volunteers was made against rabies. This vaccine was generally safe and led to the induction of neutralizing antibody that waned one year after the first vaccination ^8^. The success of mRNA vaccines has greatly benefited from the development of lipid- and polymer-based nanoparticles that protect RNA from degradation, enhanced cell uptake and improve delivery to the translational machinery. Currently, lipid nanoparticles (LNPs) are the most frequently used and effective agents for *in vivo* delivery of mRNA vaccines ^9,16,17^. Recently, the Food and Drug Administration (FDA) issued an Emergency Use Authorization (EUA) for the Pfizer-BioNTech COVID-19 (BNT162b2) vaccine (Pfizer, Inc; Philadelphia, Pennsylvania), nucleoside-modified mRNA vaccine formulated lipid nanoparticle- encoding the spike glycoprotein of SARS-CoV-2, the virus that causes coronavirus disease 2019 (COVID-19)^7^. This technology has encouraged other groups working on vaccines against cancer and viral pathogens to use the NLP-formulated mRNA platform. Recently, the Friedman group^18^ showed that nucleoside-modified mRNA in lipid nanoparticle vaccine encoding for glycoproteins gC, gD, and gE induced strong and protective immunity against acute and latent herpes simplex virus type 2 infection in mice. Indeed, and in a side-by-side experiment they compared two vaccine platforms: (1) Trivalent gC2/gD2/gE purified glycoproteins were given with adjuvants (CpG and Alum) ^19^and (2) modified mRNA encoding the 3 glycoproteins formulated in lipid nanoparticles (LNP) ^20^. The RNA was modified to increase the cellular uptake and prevent the innate immunity sensors from inhibiting the translation machinery ^21^. The mRNA-LPN vaccine demonstrated to induce effective T-follicular helper and germinal center B cell responses translated into high titers and durable antibodies responses ^22^ that outperform the glycoproteins-based vaccine in preventing HSV-1 and HSV-2 genital infection and in protecting mice and guinea pigs against intravaginal HSV-2 infection ^20^.

## Lessons Learned from Past HSV Vaccine Clinical Trials

The vaccine clinical trials produced valuable lessons that should help improve future herpes subunit vaccines. Specifically, these trials emphasize four major gaps in our current knowledge: (1) The need to incorporate protective herpes protein Ags, other than gB and gD, in the development of a future herpes therapeutic vaccine (3); (2) The need to design a vaccine strategy that induces anti-viral CD4^+^ and CD8^+^ T cell-mediated immunity (in addition to HSV-specific neutralizing antibodies) for a better protection against recurrent herpes (3). This includes exploring new adjuvants and antigen delivery systems, and (3) The need to develop a mucosal vaccine strategy that would induce strong tissue resident CD4^+^ and CD8^+^ T_RM_ cells (beside mucosal antibodies such as IgA) that would reduce virus reactivation from latently infected dorsal root ganglia (DRG) and subsequent virus shedding in the genital tract and recurrent herpetic disease. This is because of the failure of past parenteral subunit vaccines that elicit systemic immune responses against HSV-2. Although most of these vaccine research trials have not been promising, we have gained a better understanding of the correlates of protective immunity for a therapeutic HSV vaccine, forming the platform for novel combinatorial vaccine strategies against HSV.

### Phenotypic and Functionally Differential HSV-Specific Memory CD8^+^ T Cell Subsets in Asymptomatic and Symptomatic HSV Infected Individuals

**Figure 1:**
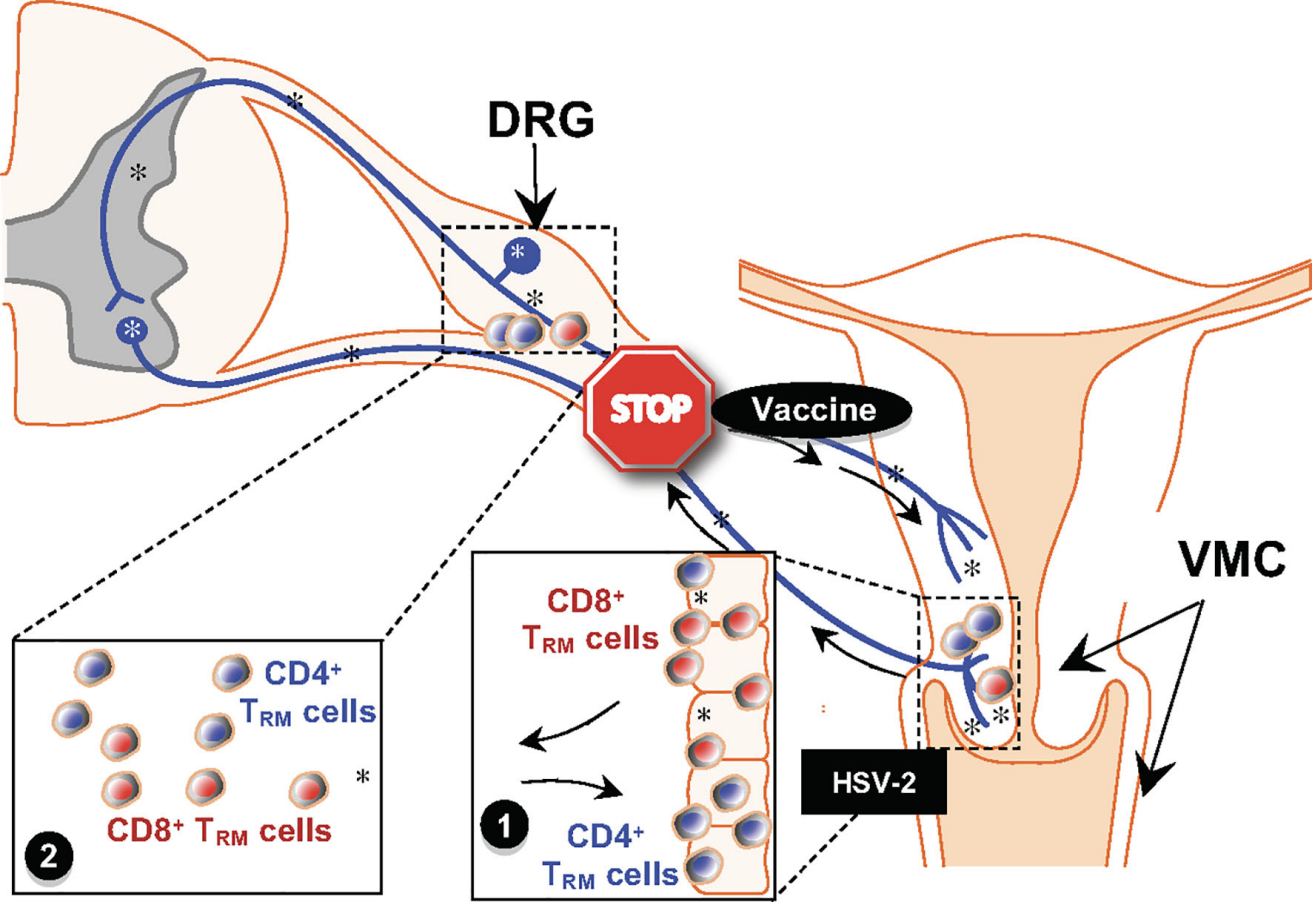
Schematic of Prime-Pull-Keep Therapeutic Vaccine (PPK Vaccine). The PPK vaccine is designed to boost Neutralizing IgG/IgA antibodies (Abs) and boost the number and function of antiviral CD4^+^ and CD8^+^ T_RM_ cells within the cervico genital muco-cutaneous [CGMC, **(1)**] and dorsal root ganglia [DRG **(2)**] tissues. The PPK vaccine is expected to help STOP the virus reactivation from latently infected DRG, virus shedding and virus replication in CGMC, thus curing or reducing recurrent genital herpes disease. *, represent virus.

Understanding the immune mechanisms by which seropositive asymptomatic individuals are protected from recurrent herpes disease is significantly important as exploiting it can elicit a T cell-based immune response in the mucosa lining the genital tract to prevent HSV acquisition. Recurrent genital herpes disease occurs following periodic reactivation of the virus that travels the axons of DRG neurons to re-infect the genital tract (GT), where lytic replication leads to herpetic lesions and transmission (15). In asymptomatic individuals (ASYMP) HSV reactivation never causes recurrent disease (16–18, 20). In symptomatic individuals (SYMP), HSV reactivation often causes painful recurrent genital disease (17, 19, 21, 22). Reports on HSV therapeutic vaccine trials have shown that both innate and adaptive immunity play an equal role in directing the right immune response to prevent disease by causing a low to no-shedding of the virus. Our research group has explored the differential immune scenarios present in asymptomatic protected individuals that gives them the natural immunity to contain recurrence of herpes. The asymptomatic and symptomatic individuals are strikingly different in their HSV-specific CD8 T memory cell immune-profile. After resolution of primary genital herpes infection, a heterogeneous pool (in terms of anatomic distribution, phenotype and fu) of HSV-specific memory CD8^+^ T cells develops (23) and can be divided into three major subsets: (**1**) effector memory CD8^+^ T cells (T_EM_) (**2**) central memory CD8^+^ T cells (T_CM_) (24) and (**3**) tissue-resident memory CD8^+^ T (T_RM_) cells. The different CD8 memory T cell subsets in HSV infection is illustrated in **Figure 1**. Regarding anatomic distribution, effector memory CD8^+^ T_EM_ cells and central memory CD8^+^ T_CM_ cells circulate between lymphoid and non-lymphoid tissues, such as the DRG and GT (24). The third subset does not enter circulation, but is instead selectively retained in infected tissues, such as DRG (25–27) and GT (25, 28), as a tissue-resident memory CD8^+^ T_RM_ cells. These CD8^+^ T_RM_ cells are poised for immediate response to reactivation from DRG (25, 29) and inhibit virus replication at GT (25). T_RM_ cells have altered T cell trafficking patterns due to the down-regulation of T cell homing molecules CD62L and CCR7 (30–34). The phenotypic profile of T_CM_ cells is CD8CD103^low^CD62L^high^ CCR7^high^. T_EM_ cells are CD8^+^CD103^low^CD62L^low^CCR7^low^. T_RM_ cells are CD8^+^CD103^high^CD62L^low^CCR7^low^CD11a^high^CD69^high^ (24, 35, 36). T_CM_ and T_EM_ cells, but not T_RM_ cells, express CD103. T_CM_ cells must proliferate and undergo differentiation for effector function (37–40). In contrast, T_EM_ and T_RM_ cells are already differentiated and poised for immediate effector function (41). We recently discovered that most HSV-specific CD8 T cells from ASYMP individuals expressed low levels of lymphoid homing markers (CD62L^low^CCR7^low^), suggesting that these T cells are predominantly of a CD8^+^ T_EM_ cell subset. In contrast, most HSV-specific CD8^+^ T cells from SYMP individuals are predominantly of T_CM_ cell subset (42). Moreover, a decline in the number and function of memory CD8^+^ T cells positively correlated with severe recurrent genital disease in SYMP individuals.

The critical role of antigen-specific CD8 T cells has been demonstrated in studies using various animal models (43, 44). We are now beginning to appreciate the differences observed in CD8 T cell memory population in symptomatic and asymptomatic HSV infected individuals, and understand the importance of stimulating tissue-resident memory T cells for prevention of HSV infection in the mouse model (44). T cell-based immunotherapeutic strategies to treat recurrent herpes infection and disease are emerging for HSV, and our laboratory has contributed significantly towards developing human asymptomatic CD8 T cell epitopes for HSV immunotherapy (20, 44, 97, 98). In the last fifteen years of vaccine development, we have succeeded in identifying new HLA-A2*01 restricted “asymptomatic” human CD4^+^ and CD8^+^ T cell epitopes from HSV-1 gB and gD glycoproteins and from HSV-1 VP11/12 and VP13/14 tegument proteins. Ocular herpes models using HLA-A2*01 restricted transgenic mouse and rabbits have shown that these asymptomatic human epitopes stimulated protective CD8 T cell responses (21, 99, 100). Presently, we are making significant headway with novel combinatorial approaches to use these epitopes as a SAPN (self-assembling protein nanoparticle) with built-in flagellin domains as a therapeutic HSV vaccine.

## Prime and Pull Vaccines Using Adenoviral Vectors Delivering Epitopes Together With T-Cell Chemokines into HSV Infected Tissues 

Chemokines are naturally produced by our immune system and could serve as safer and reliable adjuvants (101). Memory CD8^+^ T cells specific for HSV play an important role in inhibiting HSV-1 reactivation from TG and subsequent viral shedding in tears that trigger the recurrent corneal herpetic disease. The CXC chemokine ligand 10 (CXCL10)/CXC chemokine receptor 3 (CXCR3) pathways are critical in promoting T cell immunity against many viral infections (102). In a “prime and pull” strategy, a topical chemokine was applied to the genital mucosa after subcutaneous vaccination to pull HSV-specific CD8 T cells and was shown to be associated with decreased disease upon challenge with HSV-2 (103). The CXCL10/CXCR3 pathway also affects TG- and cornea-resident CD8^+^ T cell responses to recurrent ocular herpes virus infection and disease (104). Chemokines can also be co-delivered in a DNA vaccine for immunomodulation. Adenovirus-CCL21 transduced class I peptide-pulsed DC, and autologous DC-adenovirus CCL21 vaccines are currently in Phase I clinical trials for the treatment of malignant melanoma and stage IIIB-IV or recurrent non-small lung cancer respectively while XCL1 along with the *IL-2* gene (CHESAT tumor vaccine) is in a clinical trial for neuroblastoma (101). Pre-clinical studies in HSV have shown immuno-potentiation of DNA vaccines by co-delivery of chemokines such as CCR7 ligands and IL-8, RANTES delivered to the mucosa (105, 106). We are in the advent of testing multi-epitope vaccine that co-delivers chemokines using adenovirus vectors. A “Prime-Pull-Keep” Therapeutic Vaccine (PPK Vaccine) is being designed to boost Neutralizing IgG/IgA antibodies and boost the number and function of antiviral CD4^+^ and CD8^+^ T_RM_ cells within the cervico genital muco-cutaneous (CGMC) and DRG tissues. The PPK vaccine is expected to help STOP the virus reactivation from latently infected DRG, virus shedding and virus replication in CGMC, thus curing or reducing recurrent genital herpes disease (**Figure 1**).

### Laser Adjuvants

As an alternative to currently used conventional adjuvants, the chemical- and biological-free laser-adjuvant offers a well-tolerated, simple to produce method to enhance mass vaccination for widespread viral infections (107). Studies from our laboratory have reported that skin exposure of B6 mice with the FDA approved non-ablative fractional diode laser (PaloVia Laser), followed by an intradermal delivery of a HSV peptide vaccine, safely induced potent and sustained HSV-specific CD8^+^ T cells, detected in both the draining lymph nodes (DLN) and in the vaginal mucosa (VM) (108). In the vaginal mucosa of laser-treated and peptide vaccinated mice, we observed more HSV-specific effector memory CD8 T cells. Following an intravaginal HSV-2 challenge, we found decreased genital herpes lesions and increased DC infiltrates around the laser-treated skin area. These findings have important implications for the development of efficient vaccine immunization strategies against HSV-1 and HSV-2.

## Immune Checkpoint Blockade Combined With Therapeutic Herpes Vaccine

Total or partial loss of T cell function (dysfunction) occurs following repetitive HSV latent/reactivation cycles (109–111) and exposure to antigens is termed exhaustion (112) and is usually linked with expression of T cell co-inhibitory receptors: PD-1, TIM-3, LAG3 (CD223), TIGIT, PSGL-1, 2B4 (CD244), GITR, CTLA-4 (CD152), CD160, and BTLA (CD272) (113, 114). T cell dysfunction requires two signals: (**1**) T cell receptor (TCR) engaged by MHC presenting an HSV epitope (113); and a (**2**) T cell co-inhibitory receptor (e.g., PD-1) engaged by ligand (i.e., PDL-1). In humans, latent HSV in sensory ganglia is accompanied by chronic CD8 T cell infiltrates (115). A portion of viral reactivation in sensory ganglia appears to be controlled by CD8 T cell-mediated mechanisms (111, 116, 117). Recently, we compared the expression levels of eight known T cell co-inhibitory receptors on blood-derived HSV-specific CD8 T cells from symptomatic and asymptomatic HSV infected individuals and discovered that, HSV-specific CD8 T cells from symptomatic individuals expressed significantly higher levels of T cell co-inhibitory receptors like PD-1, LAG-3, TIM-3 and TIGIT (**Figure 1**). This phenotype correlated with functional exhaustion of HSV-specific CD8 T cells in symptomatic individuals with increased virus titers and severe disease. In mice, like humans, HSV-1 latently infected sensory ganglia have chronic CD8 T cell infiltrates (118). HSV-specific CD8 T cells producing IFN-γ and Granzyme B appear to suppress (or abort) induced viral reactivation in explanted mouse sensory ganglia (118, 119) and may similarly reduce detectable HSV-1 and HSV-2 reactivation *in vivo* (120–123). During acute (11 days) and latent (30 days) post-infection HSV-1infection of mice, most effector CD8 T cells from sensory ganglia simultaneously express high levels of 2 to 3 immune checkpoint receptors (e.g. PD-1 and LAG-3) (39, 111, 116, 117). This phenotype correlated with functional exhaustion of sensory ganglia-derived CD8 T cells and increased virus reactivation from infected sensory ganglia explants (39, 111, 116, 117).

Pembrolizumab and nivolumab are the first of the anti-PD-1 pathway family of checkpoint inhibitors to obtain FDA approval for the treatment of melanoma. The FDA has also granted approval of nivolumab for squamous cell lung cancer and Hodgkin lymphoma (HL), and MPDL-3280A, for bladder cancer and non–small cell lung cancer (124). From 2014-2017, the FDA approved several different anti-PD-1 mAbs opening the field of next vogues of so-called “immune checkpoint therapy mAbs” (125–127). Blocking the PD-1/PD-L1 (128–135) pathway in animal models demonstrated an improvement in CD8^+^ T cell effector function against persistent viral infections (136). Recent reports show that the natural constitutive PD-L1 expression on corneal cells impacts the HSV-1 infection of corneas. Genetic deficiency in PD-L1 using B7-H12/2 mice and the use of anti–PD-L1 blocking Ab significantly enhanced HSV-1 clearance from corneas of C57BL/6 mice mediated mainly by monocytes/macrophages (137). Based on our preliminary data of PD-L1 and GAL-9 blockade, we hypothesized that blocking PD-1, LAG-3, TIGIT and/or TIM-3 immune checkpoint pathways will help in restoring the function of HSV-specific CD8^+^ T cells in latently infected DRG and increasing efficacy and longevity of a therapeutic herpes vaccine.

## Herpes Vaccine- Safety Evaluation

Safety concerns for vaccines include: (*i*) the potential inherent toxicities of the antigen and the adjuvants, as well as potential toxicities due to interactions of the components present in the final formulation; and (*ii*) the possibility that the vaccine induces inflammatory responses that may lead to undesired toxic side effects. Some adjuvants may elicit elevated levels of proinflammatory cytokines and other mediators of toxicity, irrespective of the immune response against the antigen. Preclinical standard repeated-dose toxicology studies performed in animals will identify whether intrinsic toxicity and immunotoxicity are: (*i*) confined primarily to the sites of injection; (*ii*) caused by the delivery method (i.e., the side effects are seen in both control and vaccinated animals) or (*iii*) caused by the intended immune responses to the vaccine (i.e., side effects occur with greater frequency and severity in vaccinated animals compared to controls). (1) *Parameters for monitoring of systemic toxicity*: Toxicity studies, repeated-dose toxicity studies, address the potential for systemic toxicity including, but not limited to, the systemic effects on the immune system. A broad spectrum of information should be obtained from the toxicity study, and both in-life and postmortem data should be collected. This routinely includes careful monitoring of body weight and food consumption, body temperature, histopathology, clinical chemistry, hematology, coagulation parameters and acute phase reactants. (2) *Parameters for monitoring of local reactogenicity*: Local toxicity studies of intramuscularly administered vaccines should preferably be conducted in animals with sufficient muscle mass, (such as rabbits) to test the full human dose of the final vaccine formulation.

## Conclusions

Since most of the current HSV vaccine candidates were not promising individually in clinical trials, combinatorial vaccine approach seems to be the most appropriate in the present scenario to further advance HSV vaccine trials. Combinatorial application practically poses many problems and hence requires optimization in animal models. For example, one such approach optimized in the guinea pig model in our laboratory, is illustrated in **Figure 1**.

Results from clinical trials of the HSV vaccine indicate that it is essential to explore combinatorial approaches in the discovery of an effective therapeutic vaccine. Our long-term goal is to develop a long-lasting immunotherapeutic vaccine against genital herpes. HSV-specific CD8^+^ T cells are critical in preventing HSV reactivations from neurons of DRG and in limiting the severity of GT inflammatory lesions by reducing HSV replication (138–142). By harnessing the immune mechanisms active in seropositive asymptomatic individuals that make them “naturally” protected from recurrent herpes disease, we came up with a multiple-asymptomatic/protective epitope-based vaccine strategy, a promising HSV vaccine candidate when combined with other T cell-based immunotherapies like immune-checkpoint blockade or immunomodulation using various chemokines.

## Expert Review

▪ The latest failures of most of the clinical herpes vaccines indicate that immunotherapeutic vaccine against HSV should be efficient in eliciting antigen-specific immune responses that contain reactivation of the virus, to control both recurrent lesions and viral shedding. Our vaccine research approach is based on the understanding and harnessing of immune strategies that make the seropositive asymptomatic individuals “naturally” protected from recurrent herpes disease throughout their life. We realized that the best strategy for an effective HSV vaccine would be to elicit a T cell-based immune response that boosts HSV specific effector memory T cell functionalities in the mucosal lining to prevent HSV-1/HSV-2 acquisition/reactivation.
▪ Much remains unknown about the protective immune effector of herpes, however, improved knowledge of HSV immuno-epidemiology, and immunopathology should help guide new vaccine strategies for HSV. In the last fifteen years of vaccine development, we have succeeded in identifying many protective “asymptomatic” human CD4^+^ and CD8^+^ T cell epitopes from HSV-1 gB and gD glycoproteins and from HSV-1 VP11/12 and VP13/14 tegument proteins. We are currently progressing with novel combinatorial approaches to use these epitopes as a SAPN with built-in flagellin domains as therapeutic HSV vaccine. A Prime-Pull-Keep Therapeutic Vaccine (PPK Vaccine) is designed to boost Neutralizing IgG/IgA antibodies (Abs) and boost the number and function of antiviral CD4^+^ and CD8^+^ T_RM_ cells within the cervico genital muco-cutaneous (CGMC) and dorsal root ganglia (DRG) tissues. PPK vaccine is expected to help STOP the virus reactivation from latently infected DRG, virus shedding and virus replication in CGMC, thus curing or reducing recurrent genital herpes disease.
▪ Since most of the current HSV vaccine candidates were not promising individually in clinical trials, a combinatorial vaccine approach seems to be the most appropriate in the present scenario to further advance HSV vaccine trials. Combinatorial application practically poses many problems and hence requires optimization. We are currently optimizing these combinatorial approaches in animal models. We came up with multiple-asymptomatic/protective epitope-based vaccine strategy which will be a promising HSV vaccine candidate when combined with other T cell-based immunotherapy-like immune-checkpoint blockade or immunomodulation using various chemokines.

## Author Contributions

AC, ND, RS, SP, P-GC, and LB: conceived and designed the experiments, performed the experiments, contributed reagents, materials, and analysis tools. AC, ND, RS, SP, P-GC, LZ, HV, HC, KH-C, and LB wrote the paper. All authors contributed to the article and approved the submitted version.

## Funding

This work is supported by Public Health Service Research R01 Grants EY026103, EY019896 and EY024618 from National Eye Institute (NEI) and R21 Grant AI158060, AI150091, AI143348, AI147499, AI143326, AI138764, AI124911 and AI110902 from National Institutes of allergy and Infectious Diseases (NIAID) (to LB), and in part by The Discovery Center for Eye Research (DCER) and the Research to Prevent Blindness (RPB) grant.

## Disclaimer

The authors alone are responsible for the views expressed in this review article, and they do not necessarily represent the decisions, policy, or views of the institutions, with which they are affiliated.

## Conflict of Interest

Author HV was employed by TechImmune, LLC.

The remaining authors declare that the research was conducted in the absence of any commercial or financial relationships that could be construed as a potential conflict of interest.

## Publisher’s Note

All claims expressed in this article are solely those of the authors and do not necessarily represent those of their affiliated organizations, or those of the publisher, the editors and the reviewers. Any product that may be evaluated in this article, or claim that may be made by its manufacturer, is not guaranteed or endorsed by the publisher.
